# When Pigs Fly: Pandemic influenza enters the 21st century

**DOI:** 10.1371/journal.ppat.1008259

**Published:** 2020-03-19

**Authors:** Nídia S. Trovão, Martha I. Nelson

**Affiliations:** Fogarty International Center, National Institutes of Health, Bethesda, Maryland, United States of America; University of Michigan Medical School, UNITED STATES

## Introduction

Influenza A viruses (IAVs) are one of the most intensively studied pathogens, due to the severe global mortality and economic disruption associated with influenza pandemics [1]. In addition to annual epidemics, pandemics sporadically occur when a novel IAV host jumps to humans from an animal reservoir [2]. Wild waterfowl have long been considered the most important reservoir host and pandemic risk, but there are indications that mammals are emerging as key reservoirs. Here, we describe how the modernization of swine production during the last half century provided new opportunities for IAVs to become established in swine globally [3–5], resulting in the first influenza pandemic of swine origin in 2009 [6]. A key lesson is that the landscape of pandemic risk is not static but continuously shifting in response to demographic changes in host populations. It is therefore vital to track how transformations in the economy and global trade impact animal contact rates, disease dynamics, and pandemic risk among livestock and companion animals.

### Pigs fly?

For centuries, domestic pigs (*Sus scrofa domesticus)* were raised on traditional small-scale farms that could not sustain IAV transmission. Whereas horses and people traveled frequently within and between urban areas, causing recurrent influenza outbreaks in both species as far back as the 13th century [7], influenza was not maintained in any country’s swine population until 1918, when the Spanish influenza H1N1 pandemic virus was introduced from humans into swine in the United States [8]. The H1N1 virus circulated in US swine for most of the 20th century without substantially evolving, causing severe disease, or becoming endemic in swine in other countries.

**Fig 1:**
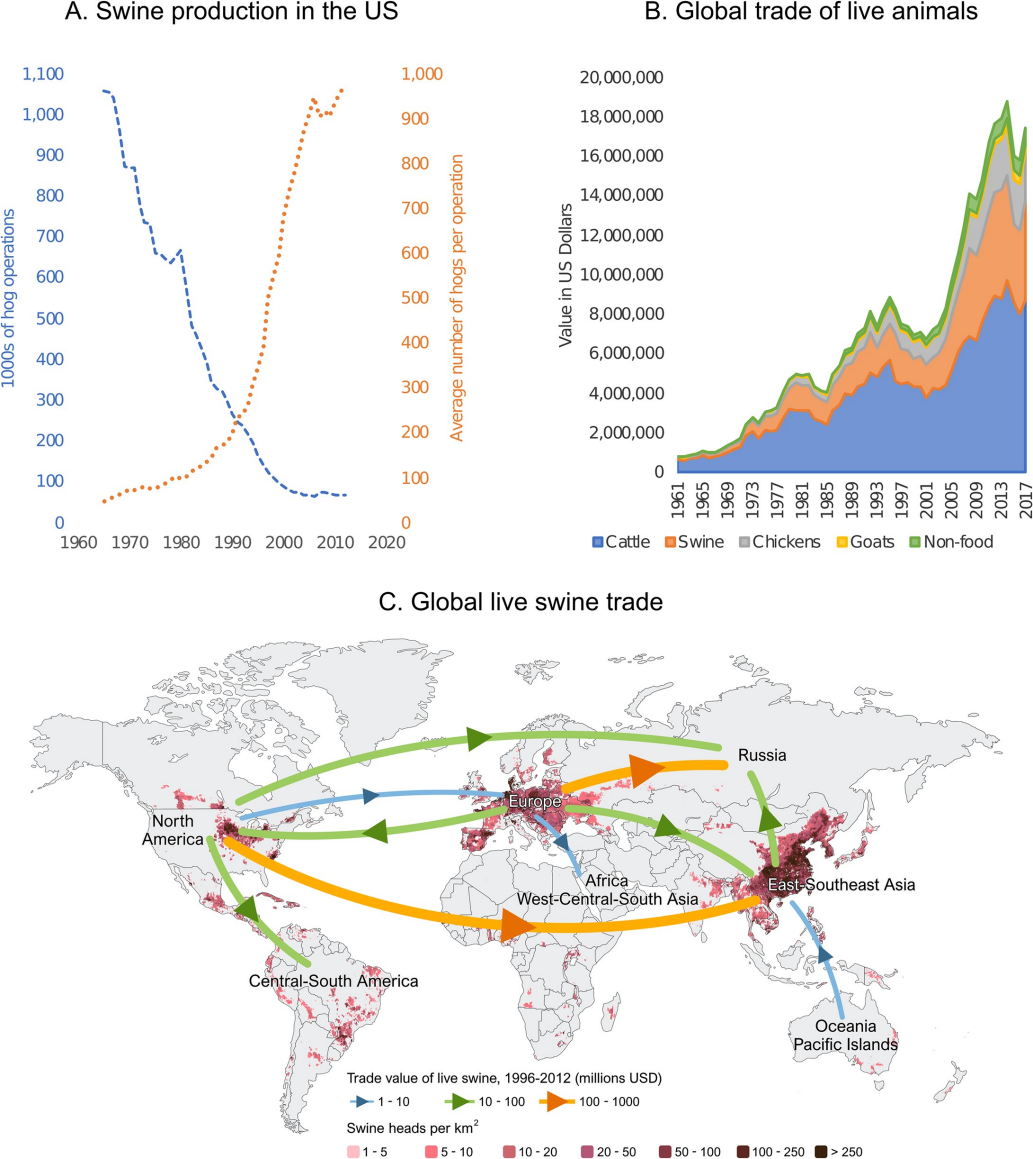
Trends in swine production. (A) Trends in consolidation of swine production in the US, 1964 to 2012 (data available from the US Department of Agriculture and the National Agricultural Statistics Service Quick Stats Database). (B) Growth of global trade (US$) of live animals between all countries, 1961 to 2017 (data available from FAOSTAT). (C) The global distribution and density of swine populations (approximately 1 billion animals) is depicted by points shaded along a gradient from light red (1 to 5 swine per km^2^) to black (more than 250 swine per km^2^). Lines with arrows depict the direction and volume of routes of trade (US$) of live swine, summarized by region and over the time period 1996 to 2012. Trade data available from United Nations Comtrade Database. Digital layers from GLW (version 2.01) [38] were downloaded from the publicly available Livestock Geo-Wiki database. FAOSTAT, Food and Agriculture Organization Statistical Database (United Nations); GLW, Gridded Livestock of the World.

In the latter decades of the 20th century, the replacement of small-scale swine farms with larger, more efficient production systems (Fig 1A) had profound effects on disease dynamics. Enhanced biosecurity improved control of important pathogens, such as hog cholera. But modern production systems often require pigs to be transported long distances between multiple locations specialized in different growth stages, facilitating the spread of IAV in swine (IAV-S; commonly known as swine flu) and other pathogens not specifically targeted for eradication. For example, many large breeding operations located in the southern US find it more efficient to transport fattening pigs to the Midwest “corn belt” than to transport the large volumes of feed back to the south. By the 2000s, trucks were transporting millions of pigs long distances across North America, facilitating the spread of IAV-S along established “swineways” [9].

**Fig 2:**
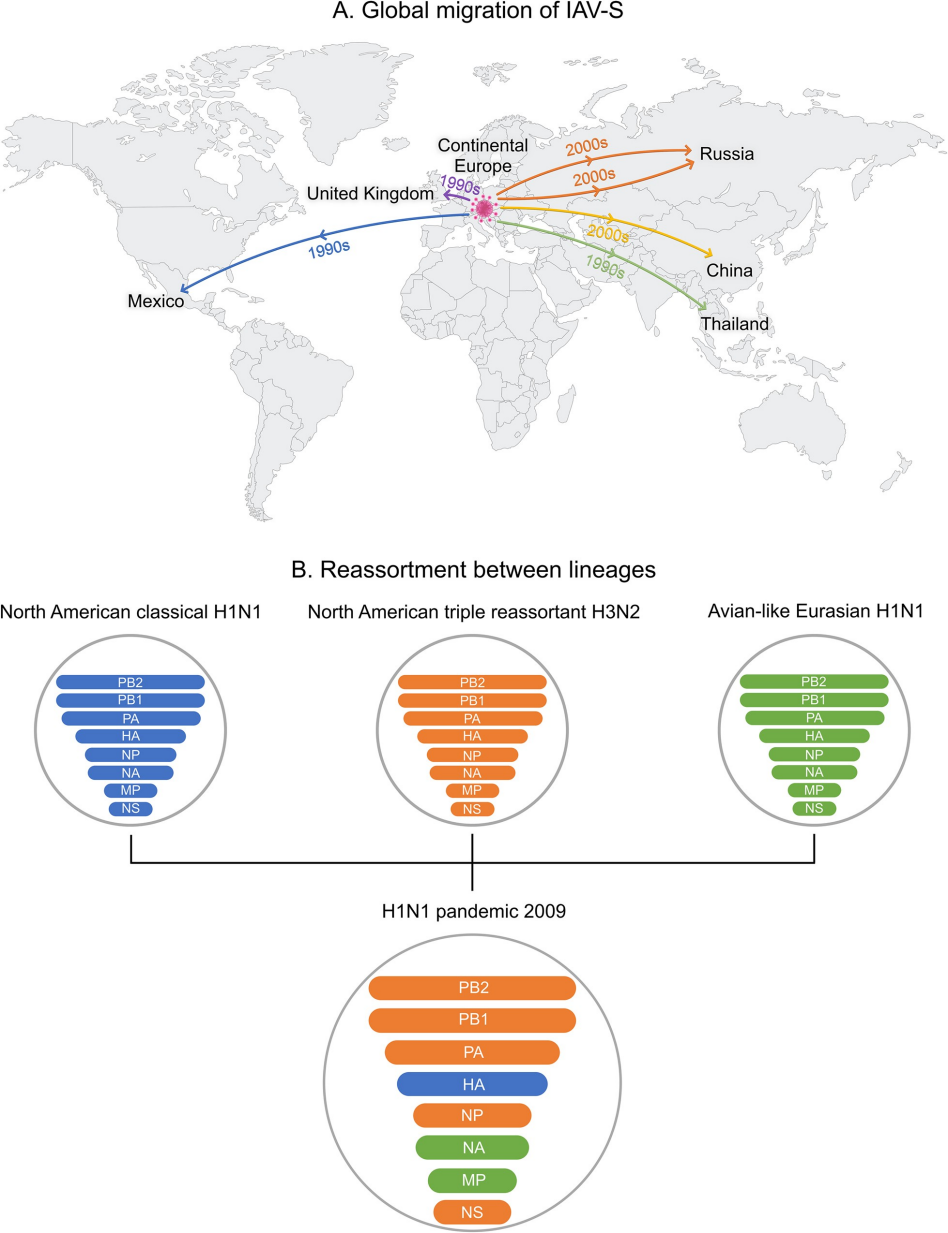
IAV-S evolution. (A) Inferred spatial movements of the major Eurasian lineage of IAV-S (avian-like Eurasian H1N1) between countries, inferred from a time-scaled MCC tree of the N1 segment. Lines represent general directions of movement inferred from available genetic data, and actual paths may differ and include unsampled locations. (B) Genomic reassortment events between the three swine lineages that produced the 2009 H1N1 pandemic virus. Horizontal bars represent the eight individual segments of the IAV genome, ordered from longest (PB2, 2,277 nucleotides) to shortest (NS, 890 nucleotides). HA, hemagglutinin; IAV, Influenza A virus; IAV-S, IAV of swine; MCC, maximum clade credibility; MP, matrix protein; NA, neuraminidase; NP, nucleoprotein; NS, nonstructural protein; PA, polymerase acidic protein; PB1, polymerase basic protein 1; PB2, polymerase basic protein 2.

In addition, pigs were flying (Fig 1B and 1C). To meet the needs of expanding middle classes for animal-sourced protein, many countries imported more productive sows (female breeding pigs) with improved genetics from North America and Europe. Imported swine must be declared free of certain pathogens, such as African swine fever (ASF) or foot-and-mouth disease (FMD). However, influenza is not routinely tested for, and many countries do not quarantine, facilitating the long-distance spread of IAV-S [3,5] (Fig 2A). Using genetic sequences of IAV-S collected in different countries over time, the direction and timing of viral movements can be inferred from the evolutionary relationships depicted on phylogenetic trees. Trees reveal how international trade of live swine in the 1990s and 2000s facilitated the long-distance dispersal of IAV-S between trade partners, shaping the global spatial distribution of IAV-S lineages that is observed today. Many countries in Asia import swine from different continents (Fig 1C), introducing multiple divergent lineages and creating nexuses for genetic diversity. By the end of the millennium, the high genetic and antigenic diversity of IAV-S made it one of the most intractable diseases for swine producers in the US and present in most swine-producing countries [4].

### How did the first pandemic virus of swine origin evolve?

It is inherently difficult to predict when and where a pandemic virus will evolve. Pandemic viruses have a rare combination of properties, including animal-origin surface proteins that are antigenically divergent from human viruses and thus evade immune detection while retaining a capacity to replicate and transmit in humans. The evolution of such a variant is facilitated by a process termed “reassortment,” in which whole segments of the IAV genome are exchanged between viruses coinfecting a host cell, rapidly repositioning genes into different genetic backgrounds (Fig 2B). Reassortant viruses are therefore more likely to evolve in locations where multiple distinct lineages cocirculate and in hosts with high capacities for reassortment, such as wild birds, poultry, and swine. The pandemic viruses of 1957 (H2N2), 1968 (H3N2), and 2009 (H1N1) were all reassortants with chimeric genomes derived from multiple lineages. The 2009 pandemic was the first to occur in the genomic era, providing large-scale sequence data to understand how globalized swine production and long-distance viral migration contributed to the evolution of a novel reassortant virus.

The 2009 pandemic vividly demonstrated both the promise and persisting limitations of outbreak investigation in the genomic era. Genetic sequencing of the first H1N1 pandemic viruses isolated from humans in April 2009 rapidly determined that the reassortant virus was comprised of three genetic lineages of swine origin (Fig 2B) [6]. However, the country of origin was unknown, due to geographical gaps in IAV-S surveillance. Many countries did not consider IAV-S an important clinical disease. To their credit, the 2009 pandemic stimulated an expansion of IAV-S research in many countries, filling gaps in our knowledge of IAV-S diversity and evolution on a global scale [10–15]. Expanded surveillance in Mexico revealed how IAV-S diversity expanded during the 1990s and 2000s, when swine were imported from Europe and the US, introducing the three IAV-S lineages that reassorted to generate the pandemic virus [16]. Going forward, trade flows can identify other countries that import live swine (and potentially IAV-S) from multiple regions and are at higher risk for generating novel reassortant viruses with pandemic potential [5].

An outstanding question is the specific circumstances by which the 2009 H1N1 virus transmitted from swine to humans. Both IAV-S sequence data and epidemiological data in humans support swine-to-human transmission occurring in Central Mexico [16,17]. However, by the time the first human cases were detected by surveillance, the virus had already diversified genetically, evidence of transmission in humans for several months [18]. There are multiple challenges to understanding the human–animal interface for IAVs: (1) There is a need for coordination between animal health and public health research, (2) the genomics of host switches are too complex to accurately predict zoonotic potential from genetic sequence alone, and (3) spillover events can be rare and difficult to detect via traditional modes of virological surveillance, particularly in developing countries.

### Pigs and humans: Who is infecting whom?

It should be noted that the human–animal interface looks quite different from the perspective of the pig. Only one IAV has successfully transmitted from swine to humans to cause a pandemic (2009) [19]. In comparison, swine are continuously experiencing pandemics of human origin. In the US alone, at least eight genetically distinct IAVs have successfully host jumped from humans to swine (reverse zoonosis). On a global scale, there have been at least 20 successful human-to-swine transmission events, defined by sustained onward transmission in swine [20]. This number could be much greater, as many countries do not routinely test for IAV-S. Tellingly, almost every country’s IAV-S population that has been genetically characterized includes viruses of human origin. Recently, viruses of human origin were identified in swine in Australia and Chile that have circulated undetected for decades [21,22].

Notably, as soon as the 2009 H1N1 pandemic virus became established in humans, the virus disseminated back to swine in at least 30 countries, spanning Africa, Asia, North and South America, Europe, and Australia [23]. In addition to seeding genetically novel viruses in swine populations globally, the human-origin viruses frequently reassort with other IAV-S lineages. The proliferation of new reassortants complicates control in swine, introducing new strains that poorly match those included in commercially available vaccines, and presents new risks for humans. As a case in point, novel IAV-S reassortants with segments derived from human-origin pandemic viruses have been associated with over 450 zoonotic infections in the US since 2011, largely in the context of agricultural fairs [24].

### What happened to bird flu?

All three pandemics of the 20th century (1918, 1957, and 1968) were of avian origin, and birds have been key sources of novel viruses in a range of other mammalian hosts, including swine, equines, canines, and phocines. For decades, the large numbers of human infections in Asia with IAVs of the H5N1 subtype and, more recently, H7N9 subtype were considered indications of impending pandemics, prompting governments to stockpile antivirals and vaccines targeting H5 and H7 antigens. H5N1 and H7N9 viruses continue to infect humans, but the absence of sustained human-to-human transmission to date underscores the difficulty of predicting pandemics, especially as large numbers of human infections and deaths are only one measure of multifaceted pandemic risk [25].

### IAV evolution never follows predictions: What will the next surprise be?

Expanded surveillance in mammalian hosts has yielded several surprises, including divergent IAVs in bats (H17 and H18) [26], a new genus of Orthomyxovirus (influenza D) in bovines [27], and the establishment of IAV in canines (CIV) during the 2000s: CIV-H3N8 in the US [28] and CIV-H3N2 in Asia [29]. An outbreak of H7N2 in felines in New York [30] suggests that other mammalian species could potentially be capable hosts for IAV transmission at certain thresholds of animal density and movement. However, IAVs have relatively low reproductive numbers (R), just above the threshold of 1 required for transmission, and require high contact rates between susceptible hosts to maintain transmission. For example, CIV in the US is maintained only in high-turnover animal shelters and dog daycares [31]. In contrast to swine, whose movements are tracked as livestock commodities and can be directly linked to disease spread [5,9], efforts to understand and predict emerging threats in companion animals are impeded by the low availability of public records and virological surveillance. Counterintuitively, shifting global attitudes towards dogs as domestic companions has facilitated the emergence of CIV. In the US, CIV-H3N2 appears to be maintained in dog daycares, which have proliferated and become mainstream. Furthermore, tThe recent introduction of CIV-H3N2 into the US spatial-temporally coincides with efforts by animal rights groups to rescue hundreds of Asian meat dogs for US adoption, although to date no direct link has been established between any rescue animals and the appearance of CIV-H3N2 in the US [32]. Dogs entering the US must provide documentation of rabies vaccination but otherwise require no disease testing or quarantine. The most pronounced change in attitudes towards dogs has been in China, where pet dogs are surging in popularity in urban areas after being banned for many decades. High rates of CIV have been observed in pets in Chinese cities [33]. It remains unclear how populations of meat dogs, strays, and pets interact and collectively contribute to CIV emergence and transmission. A diversity of swine-origin [33], human-origin [34], and reassortant IAVs have been isolated from dogs in Asia but with unknown degrees of onward transmission. High contact rates between canines and humans provides frequent opportunities for zoonotic transmission, and further research is greatly needed to understand the extent to which Asia’s rapidly expanding dog populations present a pandemic risk.

### Concluding remarks

Transformations in the movement and care of livestock and companion animal populations have altered animal contact rates, disease dynamics, and pandemic risk. At the same time, however, technological developments are underway that could enhance pandemic response. A commitment of resources has mobilized efforts to develop a universal influenza vaccine that could broadly protect humans against all animal-origin strains [35], as well as technologies to accelerate production of new vaccines during a pandemic. Harnessing new forms of digital, social, and medical claims data could detect outbreaks faster, potentially limiting spread [36]. Portable nanopore sequencing technologies are capable of tracking epidemics on location to guide intervention strategies [37]. Given these advances, it is possible to imagine a future when pandemic influenza no longer presents a threat to global health and security. However, these initiatives still face a host of technological, logistical, and market-related challenges. In the race between human ingenuity and pathogen evolution, influenza can never be underestimated.

## References

[1] van Wijhe M, Ingholt MM, Andreasen V, Simonsen L. Loose ends in the epidemiology of the 1918 Pandemic: Explaining the extreme mortality risk in young adults. Am J Epidemiol. 2018;187: 2503–2510. 10.1093/aje/kwy148 30192906 PMC7314280

[2] Webster RG, Bean WJ, Gorman OT, Chambers TM, Kawaoka Y. Evolution and ecology of influenza A viruses. Microbiol Rev. 1992;56: 152–179. 1579108 10.1128/mr.56.1.152-179.1992 PMC372859

[3] Vijaykrishna D, Smith GJD, Pybus OG, Zhu H, Bhatt S, Poon LLM, et al Long-term evolution and transmission dynamics of swine influenza A virus. Nature. 2011;473: 519–522. 10.1038/nature10004 21614079

[4] Lewis NS, Russell CA, Langat P, Anderson TK, Berger K, Bielejec F, et al The global antigenic diversity of swine influenza A viruses. Elife. 2016;5. 10.7554/eLife.12217 PMC4846380 27113719

[5] Nelson MI, Viboud C, Vincent AL, Culhane MR, Detmer SE, Wentworth DE, et al Global migration of influenza A viruses in swine. Nat Commun. 2015;6. 10.1038/ncomms7696 PMC4380236 25813399

[6] Smith GJD, Vijaykrishna D, Bahl J, Lycett SJ, Worobey M, Pybus OG, et al Origins and evolutionary genomics of the 2009 swine-origin H1N1 influenza A epidemic. Nature. 2009;459: 1122–1125. 10.1038/nature08182 19516283

[7] Morens DM, Taubenberger JK. Historical thoughts on influenza viral ecosystems, or behold a pale horse, dead dogs, failing fowl, and sick swine. Influ Other Respi Viruses. 2010;4: 327–37. 10.1111/j.1750-2659.2010.00148.x PMC3180823 20958926

[8] Koen J. A practical method for field diagnosis of swine diseases. Am J Vet Med. 1919;14: 468–470.

[9] Nelson MI, Lemey P, Tan Y, Vincent A, Lam TT-Y, Detmer S, et al Spatial dynamics of human-origin H1 influenza A virus in North American swine. PLoS Pathog. 2011;7: e1002077 10.1371/journal.ppat.1002077 21695237 PMC3111536

[10] Mathieu C, Moreno V, Retamal P, Gonzalez A, Rivera A, Fuller J, et al Pandemic (H1N1) 2009 in Breeding Turkeys, Valparaiso, Chile. Emerg Infect Dis. 2010;16: 709–711. 10.3201/eid1604.091402 20350395 PMC3321954

[11] Pereda A, Rimondi A, Cappuccio J, Sanguinetti R, Angel M, Ye J, et al Evidence of reassortment of pandemic H1N1 influenza virus in swine in Argentina: are we facing the expansion of potential epicenters of influenza emergence? Influenza Other Respi Viruses. 2011;5: 409–12. 10.1111/j.1750-2659.2011.00246.x PMC3175318 21668680

[12] Meseko CA, Odurinde OO, Olaniran BO, Heidari A, Oluwayelu DO. Pandemic influenza A/H1N1 virus incursion into Africa: countries, hosts and phylogenetic analysis. Niger Vet J. 36: 1251–1261.

[13] Holyoake PK, Kirkland PD, Davis RJ, Arzey KE, Watson J, Lunt R a, et al The first identified case of pandemic H1N1 influenza in pigs in Australia. Aust Vet J. 2011;89: 427–431. 10.1111/j.1751-0813.2011.00844.x 22008120

[14] Poonsuk S, Sangthong P, Petcharat N, Lekcharoensuk P. Genesis and genetic constellations of swine influenza viruses in Thailand. Vet Microbiol. 2013;167: 314–26. 10.1016/j.vetmic.2013.09.007 24095146

[15] Trevennec K, Leger L, Lyazrhi F, Baudon E, Cheung CY, Roger F, et al Transmission of pandemic influenza H1N1 (2009) in Vietnamese swine in 2009–2010. Influenza Other Respi Viruses. 2012;6: 348–57. 10.1111/j.1750-2659.2011.00324.x PMC3328637 22212737

[16] Mena I, Nelson MI, Quezada-Monroy F, Dutta J, Cortes-Fernández R, Lara-Puente JH, et al Origins of the 2009 H1N1 influenza pandemic in swine in Mexico. Elife. 2016;5. 10.7554/eLife.16777 PMC4957980 27350259

[17] Chowell G, Echevarría-Zuno S, Viboud C, Simonsen L, Tamerius J, Miller MA, et al Characterizing the epidemiology of the 2009 influenza A/H1N1 pandemic in Mexico. PLoS Med. 2011;8: e1000436 10.1371/journal.pmed.1000436 21629683 PMC3101203

[18] Lemey P, Suchard M, Rambaut A. Reconstructing the initial global spread of a human influenza pandemic: A Bayesian spatial-temporal model for the global spread of H1N1pdm. PLoS Curr. 2009;1: RRN1031 10.1371/currents.RRN1031 20029613 PMC2762761

[19] Garten RJ, Davis CT, Russell C a, Shu B, Lindstrom S, Balish A, et al Antigenic and genetic characteristics of swine-origin 2009 A(H1N1) influenza viruses circulating in humans. Science. 2009;325: 197–201. 10.1126/science.1176225 19465683 PMC3250984

[20] Nelson MI, Vincent AL. Reverse zoonosis of influenza to swine: new perspectives on the human-animal interface. Trends Microbiol. 2015;23: 142–53. 10.1016/j.tim.2014.12.002 25564096 PMC4348213

[21] Nelson M, Culhane MR, Rovira A, Torremorell M, Guerrero P, Norambuena J. Novel human-like influenza A viruses circulate in swine in Mexico and Chile. PLoS Curr. 2015;7. 10.1371/currents.outbreaks.c8b3207c9bad98474eca3013fa933ca6 PMC4551470 26345598

[22] Wong FYK, Donato C, Deng Y-M, Teng D, Komadina N, Baas C, et al Divergent human-origin influenza viruses detected in Australian swine populations. J Virol. 2018;92. 10.1128/JVI.00316-18 PMC6069171 29875251

[23] Nelson MI, Gramer MR, Vincent AL, Holmes EC. Global transmission of influenza viruses from humans to swine. J Gen Virol. 2012;93: 2195–2203. 10.1099/vir.0.044974-0 22791604 PMC3541789

[24] Epperson S, Jhung M, Richards S, Quinlisk P, Ball L, Moll M, et al Human infections with influenza A(H3N2) variant virus in the United States, 2011–2012. Clin Infect Dis. 2013;57: S4–S11. 10.1093/cid/cit272 23794729

[25] Trock SC, Burke SA, Cox NJ. Development of an influenza virologic risk assessment tool. Avian Dis. 2012;56: 1058–61. 10.1637/10204-041412-ResNote.1 23402136

[26] Tong S, Zhu X, Li Y, Shi M, Zhang J, Bourgeois M, et al New World bats harbor diverse influenza A viruses. PLoS Pathog. 2013;9. 10.1371/journal.ppat.1003657 PMC3794996 24130481

[27] Hause BM, Collin EA, Liu R, Huang B, Sheng Z, Lu W, et al Characterization of a novel influenza virus in cattle and swine: proposal for a new genus in the Orthomyxoviridae family. MBio. 2014;5: e00031–14. 10.1128/mBio.00031-14 24595369 PMC3958797

[28] Crawford PC, Dubovi EJ, Castleman WL, Stephenson I, Gibbs EPJ, Chen L, et al Transmission of equine influenza virus to dogs. Science. 2005;310: 482–485. 10.1126/science.1117950 16186182

[29] Song D, Kang B, Lee C, Jung K, Ha G, Kang D, et al Transmission of avian influenza virus (H3N2) to dogs. Emerg Infect Dis. 2008;14: 741–746. 10.3201/eid1405.071471 18439355 PMC2600237

[30] Hatta M, Zhong G, Gao Y, Nakajima N, Fan S, Chiba S, et al Characterization of a feline influenza A(H7N2) virus. Emerg Infect Dis. 2018;24: 75–86. 10.3201/eid2401.171240 29260686 PMC5749472

[31] Dalziel BD, Huang K, Geoghegan JL, Arinaminpathy N, Dubovi EJ, Grenfell BT, et al Contact heterogeneity, rather than transmission efficiency, limits the emergence and spread of canine influenza virus. PLoS Pathog. 2014;10: e1004455 10.1371/journal.ppat.1004455 25340642 PMC4207809

[32] Voorhees IEH, Glaser AL, Toohey-Kurth K, Newbury S, Dalziel BD, Dubovi EJ, et al Spread of canine influenza A(H3N2) virus, United States. Emerg Infect Dis. 2017;23. 10.3201/eid2312.170246 PMC5708240 28858604

[33] Chen Y, Trovão NS, Wang G, Zhao W, He P, Zhou H, et al Emergence and evolution of novel reassortant influenza a viruses in canines in southern China. MBio. 2018;9(3):e00909–18. 10.1128/mBio.00909-18 29871917 PMC5989073

[34] Song D, Moon H-J, An D-J, Jeoung H-Y, Kim H, Yeom M-J, et al A novel reassortant canine H3N1 influenza virus between pandemic H1N1 and canine H3N2 influenza viruses in Korea. J Gen Virol. 2012;93: 551–4. 10.1099/vir.0.037739-0 22131311 PMC3352354

[35] Erbelding EJ, Post DJ, Stemmy EJ, Roberts PC, Augustine AD, Ferguson S, et al A Universal influenza vaccine: the strategic plan for the National Institute of Allergy and Infectious Diseases. J Infect Dis. 2018;218: 347–354. 10.1093/infdis/jiy103 29506129 PMC6279170

[36] Simonsen L, Gog JR, Olson D, Viboud C. Infectious disease surveillance in the Big Data era: towards faster and locally relevant systems. J Infect Dis. 2016;214: S380–S385. 10.1093/infdis/jiw376 28830112 PMC5144901

[37] Quick J, Loman NJ, Duraffour S, Simpson JT, Severi E, Cowley L, et al Real-time, portable genome sequencing for Ebola surveillance. Nature. 2016;530: 228–232. 10.1038/nature16996 26840485 PMC4817224

[38] Robinson TP, Wint GRW, Conchedda G, Van Boeckel TP, Ercoli V, Palamara E, et al Mapping the global distribution of livestock. PLoS ONE. 2014;9: e96084 10.1371/journal.pone.0096084 24875496 PMC4038494

